# Methodological review: quality of randomized controlled trials in health literacy

**DOI:** 10.1186/s12913-016-1479-2

**Published:** 2016-07-11

**Authors:** Julii Brainard, Stephanie Howard Wilsher, Charlotte Salter, Yoon Kong Loke

**Affiliations:** Norwich Medical School, University of East Anglia, Norwich, NR4 7TJ UK

**Keywords:** Health literacy, Randomised controlled trial, Public health, Risk of Bias, Quality of Evidence

## Abstract

**Background:**

The growing move towards patient-centred care has led to substantial research into improving the health literacy skills of patients and members of the public. Hence, there is a pressing need to assess the methodology used in contemporary randomized controlled trials (RCTs) of interventions directed at health literacy, in particular the quality (risk of bias), and the types of outcomes reported.

**Methods:**

We conducted a systematic database search for RCTs involving interventions directed at health literacy in adults, published from 2009 to 2014. The Cochrane Risk of Bias tool was used to assess quality of RCT implementation. We also checked the sample size calculation for primary outcomes. Reported evidence of efficacy (statistical significance) was extracted for intervention outcomes in any of three domains of effect: knowledge, behaviour, health status. Demographics of intervention participants were also extracted, including socioeconomic status.

**Results:**

We found areas of methodological strength (good randomization and allocation concealment), but areas of weakness regarding blinding of participants, people delivering the intervention and outcomes assessors. Substantial attrition (losses by monitoring time point) was seen in a third of RCTs, potentially leading to insufficient power to obtain precise estimates of intervention effect on primary outcomes. Most RCTs showed that the health literacy interventions had some beneficial effect on knowledge outcomes, but this was typically for less than 3 months after intervention end. There were far fewer reports of significant improvements in substantive patient-oriented outcomes, such as beneficial effects on behavioural change or health (clinical) status. Most RCTs featured participants from vulnerable populations.

**Conclusions:**

Our evaluation shows that health literacy trial design, conduct and reporting could be considerably improved, particularly by reducing attrition and obtaining longer follow-up. More meaningful RCTs would also result if health literacy trials were designed with public and patient involvement to focus on clinically important patient-oriented outcomes, rather than just knowledge, behaviour or skills in isolation.

**Electronic supplementary material:**

The online version of this article (doi:10.1186/s12913-016-1479-2) contains supplementary material, which is available to authorized users.

## Background

As populations age and live longer with (often multiple) chronic conditions and healthcare becomes ever more complex and fragmented there is widespread recognition that many people lack the knowledge and skills required to best manage their own health needs, particularly in response to medical advice. This set of useful capacities are often described under the umbrella term of *Health Literacy*, which may also be defined as *the degree to which individuals have the capacity to obtain, process, understand and use basic health information and services needed to make appropriate health decisions* [1].

Inadequate health literacy (HL) is strongly associated with poor health outcomes in populations or individuals [2], and this lack of knowledge, skills and capacity has been estimated to add an extra 3-5 % to national health care budgets [3]. Disadvantaged groups are at greater risk of having relatively low health literacy, which also makes it a social justice issue [4]. Addressing health literacy is essential to maximise the potential positive benefits of other popular initiatives in modern medicine, such as patient-centred care [5], and shared decision-making [6]. It has even been argued that, with regard to verifying efficacy, addressing “health literacy is as important as randomization and statistical analyses in the research design of educational interventions” [7].

Hence, there is growing focus on efforts to improve health literacy in the hope that this will also result in better patient outcomes and wider societal benefits. The policy agenda of improving population health literacy has been embraced by governments [8–10] and professional associations [1, 11, 12]. Academic research on HL is increasingly prominent with growing numbers of published interventional studies. Systematic reviews of health literacy interventions [2, 13] report overall modestly positive impacts, but there appears to be some variation in efficacy, with inconsistent long-term improvements of health literacy skills in individuals. More importantly, reviews also repeatedly comment that there is a lack of high quality evidence about which strategies are most effective [2, 9, 14].

Recommendations have emerged that there should be comprehensive evaluation measures with development of experimental designs that better support the research outcomes in relation to health literacy interventions [9, 15]. The lack of robust evidence creates difficulties in evidence grading and policy development [9, 16] because decisions on use of HL interventions should be backed up by good evidence of benefit, with low risk of bias from methodologically rigorous trials.

Consequently, there is a pressing need to assess the methodology used in contemporary HL interventions, in particular the quality (or risk of bias [17]) within randomized controlled trials (RCTs). Identification of methodological strengths and limitations would raise awareness of design limitations, and stimulate development of rigorous and perhaps innovative strategies for high quality HL RCTs. Hence, we conducted a methodological review of recent RCTs describing HL interventions in order to determine the methodological quality and types of outcomes evaluated. Many attributes of quality of trial conduct were considered (detailed below). Also, because health literacy can be a social justice issue, it may be argued that HL interventions should prioritise targeting vulnerable individuals. Yet, other research suggests that vulnerable groups are under-represented in both health-management programmes [18] and clinical research trials [19]. Therefore, we also recorded demographic characteristics of patients in each study, in order to determine the types of populations involved in HL trials.

## Methods

### Selection criteria

We aimed to assess contemporary (publication year 2009–2014) randomized controlled trials of health literacy interventions in the published literature. The selection criteria were:

- Participants were adults age 18 years and above
- Must describe an intervention that applies health literacy concepts (compatible with the definition of health literacy as stated earlier [1]), or uses low literacy tools to improve health-related outcomes. We only included articles where health literacy was explicitly stated and a key component in how the intervention was designed
- Intervention target must be same person that the benefits are measured for
- Outcomes must be measured in terms of improvement in three areas, that we broadly summarise as ‘knowledge’, ‘behaviour’ or ‘patient well-being/health’. These are described in much more detail below.

We excluded studies that featured children (under 18 years old) as targets or indirect beneficiaries, or that were applied within formal educational programmes leading to qualifications. Studies that looked at associations or correlation rather than effect of the interventions, (for instance, a health promotion project that made a link between outcomes and pre-existing health literacy levels in participants), were excluded. We excluded abstracts because we felt that there would not be sufficient space for authors to report full methodological details.

### Search strategy

We searched Pubmed on 1 December 2014, using a validated algorithm [20] that gives the best balance between specificity and sensitivity:

- (randomized controlled trial[Publication Type] OR randomized[Title/Abstract]) AND
- ("health-literacy"[All Fields] OR "health-literacy"[MeSH terms])

Those search terms were duplicated as closely as permissible to find additional articles in the following sources (see Additional file 1: Table S1 for specific search terms): Embase, Cochrane central, Cinahl, Psychinfo and (United States National Cancer Institute) Research-tested Intervention Programs (RTIPS). There were no restrictions for language or country.

### Study screening and data extraction

Abstracts and titles were independently duplicate-screened to remove citations that failed to meet the inclusion criteria (listed above under selection criteria). The full text version of the remaining potentially relevant articles that passed through abstract + title screening was read by two screeners (JB, YKL) to confirm eligibility.

Data were independently extracted by at least two reviewers (JB, SHW, YKL) from all remaining eligible articles, using a customised data extraction form that recorded bibliographic details, study location(s) and funder(s), patient demographics, number of patients in each trial arm and outcomes. Disagreements at all stages were resolved by discussion or using referral to a third reviewer (YKL or CS).

### Assessment of trial quality

Relevant to statistical significance, we noted whether recruitment targets were calculated to ensure that statistical calculations were adequately powered for the stated “primary” outcome, and whether actual recruitment subsequently met stated targets.

We used the Cochrane Risk of Bias tool to assess quality of RCT implementation and reporting [17]. Risk of bias (RoB) for *random sequence generation* was assessed as low if the authors indicated that a random number generator was used. Low RoB for *allocation concealment* resulted if the authors explained clearly how both participants and investigators were masked to group assignment at the moment of allocation. RoB for trial *performance* or *detection* was only assessed as low if investigators were masked during intervention delivery (performance) and monitoring (detection). Low RoB for *attrition* was assessed if total loss was below 20 % between intervention start and last monitoring date.

In order to detect the possibility of bias due to *selective reporting* bias, we recorded and compared the pre-specified outcomes in the Methods section against the list of outcomes that were actually reported in the Results section (see Additional file 1: Item S2). This was intended to allow us to judge the possibility of any missing outcome data or subsequent addition of *post-hoc* subgroup analyses based on presence or absence of statistically significant findings. Previous comparisons of protocols or registry entries of published reports for RCTs suggested that it is not unusual for primary outcomes in final reports to vary from those that were pre-specified [21].

### Evidence of efficacy

We categorized outcomes of the interventions into one of three categories: knowledge, behaviour or health-related (K, B or H), by drawing on relationships between knowledge and behaviour with health outcome improvements that have been identified by others [22, 23]. *Knowledge* encompassed both patient understanding and awareness of the disease course and aims/strategies of treatment (e.g., complications of high blood pressure and drugs that can control it). *Behaviour* changes included all actions that may improve health (such as using smartphone reminders for timing of blood pressure medication) or improvement in skills, self-efficacy, readiness to change or intentions. *Health* outcomes were defined as clinical measures in patients such as reduction in distress (or better quality of life), weight loss or improved disease indicators (such as lower blood pressure or normal laboratory test results). These changes could be assessed subjectively or objectively. We were concerned to focus on outcomes that indicate independent acquisition of understanding or skills, shown through sustained behaviour or improvements in experienced health. We did not aim to analyse or categorize some outcomes such as decisions to take-up cancer screening tests [24] and accuracy in following simultaneous instructor-guided instructions [25], because these involved complex, multifaceted components, subjective value judgement or manual dexterity skills in artificial test settings. We extracted information on the stated RCT outcomes in KBH categories whether or not they were significantly better (p ≤ 0.05) for the intervention over control arm.

### Demographics of participants and other study aspects

We recorded participant characteristics, including socio-economic traits: mean age, gender balance and percentage of trial participants that were ethnic minorities (within that country), low income (defined as income < US $20,000/year), or low education (less than 12 years or US high school diploma equivalent (GED). We also noted the duration of monitoring post intervention.

## Results

**Fig. 1:**
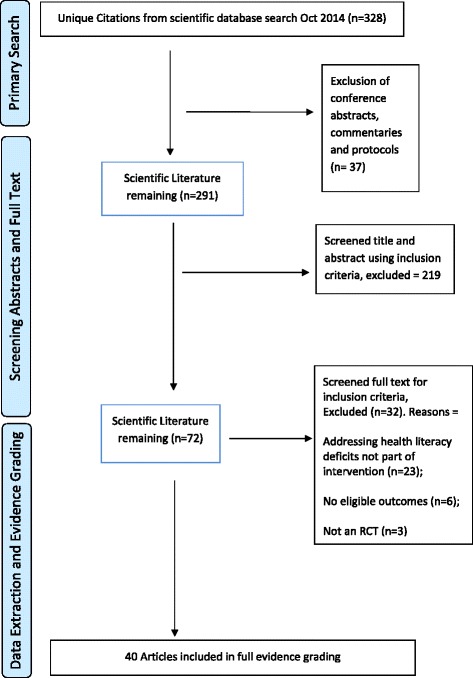
Flow diagram of study selection

We identified 328 potentially relevant RCT reports from the searches, and after screening, we performed data extraction on 40 included papers (references numbered 24, 25, 28–48 and 60–76). The study selection process is shown in Fig. 1.

### Study characteristics

The number of RCTs published each year increased over time, starting with three papers in 2009, four papers from 2010, five articles from 2011, seven published in 2012, 13 in 2013 and eight papers in 2014. Typically, interventions were educational in approach – full details of the interventions, control arms, and the relationship to health literacy are listed in Additional file 1: Table S3. Twelve papers addressed aspects of managing chronic illness (most often Type 2 diabetes). Correct administration of medication and aspects of mental health problems were each the focus of seven RCTs. Nutrition choices were the focus of six articles. Other trials covered cancer screening, physical activity, patient-provider communication, sterilisation choices and preventing cardiovascular disease. Country locations were 82.5 % USA, 15 % Australia, and one study in Iran (2.5 %). Funding mostly came from national government bodies. 55 % were solely government funded, 15 % of articles mentioned funding from only charitable groups. 12 % of articles listed a mix of government and charity funders, while in 18 % of studies the funding was other (usually insurance companies or academic institutions) or unclear.

### Demographics of participants and other study aspects

Table 1 shows demographic information for study participants, as well as duration of monitoring period and validity of health literacy instruments used (if any). Low income and ethnic minority individuals were mostly over-represented, which is probably desirable because they are especially vulnerable both for poorer health outcomes and for likelihood that low health literacy will increase risk of poor health outcomes [4]. This representation pattern may reflect priorities of the funding bodies (mostly government institutions and charities). The percentage of participants with low educational attainment, and the percentage with low or inadequate HL as calculated by formal instruments, is about average for the USA and Australia [26, 27]. It is not surprising that participants were mostly female and mostly middle aged or older (this group tends to be strongly represented in educational interventions) [18]. Targeting people age 50+ is desirable because health literacy tends to decline with age and people age 65+ are a distinct at-risk group for low health literacy [16].

**Table 1:** Average statistics for these attributes of target participants in the 40 selected RCTs

% Who are not the dominant ethnic group within that country *N* = 38	% Low income (<= US $20 k/year) *N* = 18	% With low or inadequate HL *N* = 23	% with < 12 years of eductn or GED *N* = 33	Mean age (yrs) *N* = 40	Gender % F *N* = 39	Latest observation point after intervention start (weeks) *N* = 40	% clearly used a validated HL instrument *N* = 40
57.7 (23–95)	54.7 (30–76)	43.3 (23–61)	36.5 (3–64)	52 (45–60)	63.7 (60–77)	23 (2–26)	73

*N* = number of studies where data were available. Interquartile 25th–75th percentile range in parentheses

### Methodological characteristics/Trial quality

Many (65 %) of the RCTs reported power calculations for a target sample size on their primary outcome. However, relatively few (20 % of the total 40 studies) retained enough participants until the final monitoring date so as to be adequately powered to detect a meaningful effect. Therefore, most studies were, in at least some of their results, under-powered, thus creating uncertainty and imprecision in estimates of the intervention’s effect.

Table 2 summarises the distribution of Risk of Bias decisions for the 40 RCTs. Most (73 %) had low bias for random sequence generation. About half (53 %) had low bias for allocation concealment. About a fifth (23 %) had low performance bias, roughly one third had low detection bias (35 %) and two thirds had low attrition bias (68 %). Unclear reporting was a common problem in these RCTs. Risk of bias was recorded as unclear for 29–41 % of studies in the first four domains but the number of participants in each arm from initiation to final monitoring point was always clearly reported.

**Table 2:** Percentage of studies with stated Risk of Bias in each domain (*n* = 40)

	Random sequence generation (selection bias)	Allocation concealment (selection bias)	Blinding of participants and personnel bias (performance bias)	Blinding of outcome assessment (detection bias)	Incomplete outcome data (attrition bias)
High	0 %	15 %	38 %	25 %	33 %
Unclear	28 %	33 %	40 %	40 %	0 %
Low	73 %	53 %	23 %	35 %	68 %

### Selective reporting bias

Eight studies reported unexpected (secondary) outcomes: that is, those not specified in the methods section: three were on knowledge and use of the intervention materials [28–30], while significant results for knowledge, different HL levels, and use of the intervention were reported in three studies [31–33]. One report highlighted significant interactions between patient performance on hypertension knowledge and the study interviewer [34]. Another found demographic factors such as income, HL age and ethnicity were significant or almost significant predictors of (medication adherence) error rate [35]. Protocols were available for only six of the 40 included studies [36–41]. Of these, only three final trial reports deviated from protocol (arguably minor deviations). Duncan et al. [37] omitted a secondary outcome (weight change), Freed et al. [38] changed primary outcome from “comprehension” to “word recognition”, while Gulliver et al. [39] reported on stigma rather than “attitude” as described in the protocol.

### Study outcomes

Table 3 describes the outcomes reported by the trials, outcomes were grouped together where very similar. In Table 3, the numbers to the left indicate how many studies reported at least one intervention benefit in K, B or H areas (statistically significant at p ≤ 0.05). The largest groups of outcomes related to mental health issues (mostly depression), nutrition, diabetes self-management and medication adherence.

**Table 3:** List of outcomes reported in health literacy RCTs published 2010–2014

Focus of the study	#SS	Knowledge outcomes	#SS	Behavioural outcomes	#SS	Health outcomes
Medication-related	4	How to take medication [25, 35, 42, 60]	1 1	Self-reported medication adherence [28, 61–64] Observed medication adherence [31, 65, 66]	0	Number of clinically important medication errors [67]
Health-related	4	Diabetes knowledge or health literacy [43, 45, 62, 68]	1	Glucose-self-monitoring [45]	0 1	Improvement in HbA1C [36, 68] Viral load (HIV) [31]
	1	Recall of cancer screening knowledge [38, 69]				
	1	Breast cancer knowledge [24]	0	Increase in discussion about screening decisions [41]	0	Overall health status [44, 45, 63, 65, 70]
	1	Sterilisation knowledge [30]	0	Improved communication with health care professionals [65, 69]		
	1	Hypertension knowledge [34]			0	Systolic BP [28, 36]
	1	Recognition of heart attack symptoms [71]	1	Improved inhaler technique (COPD) [72]		
	0	Knowledge of cardiovascular disease or stroke and their risk factors [28, 71]	1	Creation of self-management plan for asthma [73]	1	Asthma-impact on quality of life indicators [73]
	0	Recognition of stroke symptoms [71]	3	Self-efficacy [45, 46, 63, 64]	0	Improvement in LDL-cholesterol [36]
Mental health related	4	Depression or mental health knowledge and literacy [32, 33, 39, 40, 44, 74]	5	Attitudes, intentions, stigma or behaviour about seeking support for mental health issues, especially depression [32, 33, 39, 40, 44, 74]	1 0	Reduction in emotional distress (including anxiety) [32, 44, 46, 68] Overall mental health status [48, 63]
					2	Decision-conflict [24, 75]
Behaviour/lifestyle-related	1	Knowledge of healthy nutrition [29, 37, 76]	3	Nutritional choices and attitudes [28, 37, 45, 62, 68, 76, 77]		
	1	Understanding labels [29]	2	Increase in exercise [28, 37, 45, 47, 48, 68]	1	Weight loss [28]
	0	Recall of healthy lifestyle advice [65]	0	Attempts to comply with multifactor health lifestyle advice [65]	0	Folate B12 and homocysteine concentration in blood [48]
			0	Reduced smoking [28]		
			0	Reduced alcohol consumption [32]		
			0	Appointment keeping [63]		
			1	Home safety actions [46]		

#SS = Number of studies that reported at least one intervention benefit (statistically significant at *p* ≤ 0.05) for stated outcome

### Evidence of efficacy

The underlying heterogeneity of the specific outcome measures and the small pool of articles addressing each outcome make formal meta-analysis inappropriate but some observations may be made about groups of related outcomes in Table 3. Measurement of the intervention’s effects on knowledge was far more frequent than behaviour change or improved health indicators. For instance, aspects of medication adherence were a common theme. All studies to improve medication knowledge reported success, but only 2 of 8 studies that attempted to improve adherence behaviour were successful, and clinically important medication errors were not avoided in another study. Similarly, diabetes knowledge or literacy improved in 4/4 studies, but HbA1C measures in blood improved in none of three studies. An exception to this trend is with regard to attitudes towards mental illness (especially depression). Depression knowledge and literacy improved in most interventions (4/5 trials) while less stigma and increased willingness to seek or offer help for people suffering depression was also often reported in the intervention group (which we deemed a behavioural change, reported in 5/6 studies). Such a readiness to change or other improvement in aspects of self-efficacy was not as common for other health topics.

Table 4 summaries how many distinct outcomes each RCT reported (within the KBH categories) that were statistically significant (better) for intervention over control arms, at the latest monitoring time point for which data were available. 42.5 % (17 of the 40 studies) had no improvement in any knowledge outcome at the last monitoring point. 67 % (27) had no improvement in behaviour outcomes, 92 % (37 of the 40) had no health outcome differences between the intervention and control arm. Some studies reported up to 3 benefits in knowledge [25, 39, 42–44] or behaviour [45–47] outcomes, however. There were 31 trials that specified at least one primary outcome, of which statistically significant effects of the intervention were reported in 17.

**Table 4:** Percentage of studies with improvement in given number of individual indicators in each KBH areas

Area	None	1 outcome	2 outcomes	3 outcomes	Totals
Knowledge	42.5 %	32.5 %	12.5 %	12.5 %	100 %
Behaviour	67.5 %	15 %	10 %	7.5 %	100 %
Health	92.5 %	5 %	2.5 %	0 %	100 %

Most RCTs (29/40 = 72.5 %) reported improvements in at least one of the KBH areas. Conversely, 27.5 % (11/40) of studies recorded no significant between group differences for any of the KBH outcomes, at the final monitoring point.

### Follow-up analyses

Sixteen studies (40 %) had no follow-up analysis (monitoring of impacts after day of intervention end). Nine studies (22.5 %) had follow-up that was 1–4 weeks after the intervention finished, and nine further trials followed up between 8 weeks and 6-months post-intervention. Six trials (15 %) had followup > 6 months (up to 12 months). With regard to trial quality, it is noticeable that the six trials that monitored for > 6 months all reported at least one statistically significant outcome in the KBH areas. Length of follow-up period otherwise appeared to have no association with KBH results. Some RCTs reported transient benefits soon after the intervention, but between-group differences were not found at the final monitoring date [29, 31, 33, 45, 48].

## Discussion

We found areas of strengths (good randomization and allocation concealment), but areas of weakness regarding blinding of participants, people delivering the intervention and outcomes assessors. Substantial attrition (losses by monitoring time point) was seen in a third of RCTs. This creates difficulty in interpreting findings of lack of benefit, because it may be due to inadequate power, rather genuine absence of efficacy. These important limitations undermine the validity of actual recent RCTs in health literacy.

Blinding to prevent performance and detection bias in educational or behaviour-related RCTs is difficult but not impossible. Attrition bias is much harder for trialists to control. Most of our reviewed RCTs had recruitment targets guided by formal power calculations, but most of these studies also failed to retain as many participants (to the final monitoring time point) as their power calculations required. It was not clear from these RCTs if there were commonly avoidable reasons for attrition rates. As a short term solution, it may be best that recruitment targets are raised for health literacy RCTs in order to ensure adequately powered results. In general, more research is needed about how to ensure high recruitment to RCTs [49].

Evidence of reporting bias was not generally found, but this may reflect *post-hoc* addition or omission of intended study outcomes. Also, some studies only achieved statistical significance for outcomes by looking for improvements within subgroups, particularly individuals with the lowest levels of initial health literacy [28, 29, 43]. Subgroup analysis is helpful scientifically and justified given the potentially diverse educational needs of different population groups, but such findings need to be cautiously interpreted. 45 % of RCTs (14 of the 31 studies that specified primary outcomes) found no intervention benefits in the primary outcome(s) at their final monitoring time point.

We are concerned by the predominance of short-term, knowledge based outcomes in health literacy RCTs. Only 15 % of our included RCTs followed up beyond 6 months. The short time scale of many RCTs does not inform which strategies are most sustainable, and therefore may be most cost-effective and ultimately lead to maximum patient benefits. Hence it is perhaps not surprisingly that our included health literacy RCTs had modest success at changing clinical outcomes (= tangible improvement in actual patient health). Ioannidis [50] concluded that too many RCTs are problematic in execution, stating that many

*“…simply represent wasted effort because the questions they ask and the comparisons and outcomes they choose to study are clinically irrelevant. Looking at the many thousands of clinical trials launched annually, this irrelevance may be actually the biggest source of waste in randomized controlled trials*…”

Ioannidis stated that it was regrettable that few trials in the published literature are guided from inception by patient-centred outcomes [50], even though it has been accepted for some time that an evidenced-based approach to patient care should be informed by “*what is meaningful and valuable to the individual patient*” [51]. Instead, research tends to focus on academic and clinical researcher-preferenced knowledge or skills tests, ignoring the social, cultural and economic contexts in which patients live. Health literacy trials frequently focus on the individual as problematic rather than the larger context and the demands placed on patients by complex modern healthcare systems [52]. Detailed, well thought-out patient participatory process evaluations within trials are rare, which means that patient experience and understanding of important factors such as patient-clinician relationships are missed [53]. Health promotion efforts, including health literacy RCTs, are prone to an underlying bias that if people are told and trained what to do, they will both do it and become healthier, when in reality the relationship between knowledge, behaviour and health outcome is complex and highly personal [54, 55]. A more sustainable strategy to effective promotion of healthy behaviour (including design of RCTs designed to address health literacy deficits) may be to involve patients in intervention design and implementation, as a form of patient-centred care [51] that considers many contextual aspects of barriers to knowledge, healthier behaviour choices & skills acquisition. Otherwise, it seems likely that without the engagement of the patient and their family, especially when increasing multimorbidity is involved, such trials will fail to reflect the reality of managing health for the individual, and weak or no positive outcomes beyond the duration of the trial are likely to follow.

### Limitations of our review

We included relatively recent trials (published 2010-2014) to provide a snapshot of what is actually happening rather than create a historical perspective. Nevertheless, poor quality reporting meant that in many instances, we had to record Risk of bias (RoB) domains as unclear. Such research is obviously still developing and it may be that the quality of HL research has not yet advanced to the quality standard that Cochrane RoB tools demand, or perhaps that Cochrane RoB tools are not fully suited to these types of complex or educational interventions.

We report only on published literature; there may well be unpublished trials that are of different quality. It is widely recognised that trials without significant results are less likely to be published [50], and that over-representation of successful studies undermines the reliability of scientific conclusions [56]. Almost all of the literature we found was from English-speaking countries, particularly from the USA, and to some extent the format, priorities and successes of these interventions must be biased by cultural influences.

## Conclusions

The implications of our review are that while trials in HL are of growing interest, they may pose difficult methodological challenges because of the nature of the topic and its interventions. Our methodological evaluation shows that health literacy trial design, conduct and reporting could be considerably improved. To support such development, trialists can refer to many existing guidelines on good methodological practice for implementing and reporting RCTs: eg CONSORT 2010 statement [57] or COMET initiative [58].

Assessing quality of evidence (as we have done) is an essential pre-requisite before selecting and implementing interventions for patient benefit [59]. We can also recommend: Health literacy trials should be informed by inclusion of patient-centred health outcomes at their inception, design, delivery and evaluation stages. Without this, any findings have the potential to be meaningless.

## Abbreviations

B, behaviour; BP, blood pressure; COMET, core outcome measures in effectiveness trials; CONSORT, consolidated standards of reporting trials; GED, general educational development (test); H, health (clinical outcomes); HbA1C, haemoglobin A1C; HIV, human immunodeficiency virus; HL, health literacy; K, knowledge; KBH, knowledge, behaviour or health-related (outcomes); RCT, randomized controlled trial; RoB, risk of bias; US or USA, United States of America; yr, year

## Additional file

Additional file 1: Table S1. Search phrases and results. Item S2: Data extraction form. Table S3: Study characteristics. (DOCX 82 kb)

## References

[1] Kindig DA, Panzer AM, Nielsen-Bohlman L. Health Literacy: A Prescription to End Confusion. Washington D.C.: National Academies Press; 2004. 25009856

[2] Berkman ND, Sheridan SL, Donahue KE, Halpern DJ, Viera A, Crotty K, Holland A, Brasure M, Lohr KN, Harden E (2011). Health literacy interventions and outcomes: an updated systematic review. Evid Rep Technol Assess.

[3] Eichler K, Wieser S, Brügger U (2009). The costs of limited health literacy: a systematic review. Int J Public Health.

[4] Logan RA, Wong WF, Villaire M, Daus G, Parnell TA, Willis E, Paasche-Orlow MK (2015). Health literacy: a necessary element for achieving health equity.

[5] Paasche‐Orlow MK, Schillinger D, Greene SM, Wagner EH (2006). How health care systems can begin to address the challenge of limited literacy. J Gen Intern Med.

[6] Edwards A, Elwyn G. Shared decision-making in health care: Achieving evidence-based patient choice. Oxford University Press; 2009.

[7] Zeni MB (2012). Systematic review of health literacy in Cochrane database studies on paediatric asthma educational interventions: searching beyond rigorous design. Int J Evid Based Healthc.

[8] Australian Commission on Safety and Quality in Health Care. Health Literacy: Taking action to improve safety and quality. Sydney NSW: Commonwealth of Australia. 2014.

[9] Heijmans M, Uiters E, Rose T, Hofstede J, Deville W, van der Heide I, Boshuisen H, Rademaker J. Study on sound evidence for a better understanding of health literacy in the European Union. Brussels: European Commission. 2015.

[10] Health UDo, Services H, Prevention OoD, Promotion H. National action plan to improve health literacy. Washington, DC: Department of Health and Human Services; 2010.

[11] Rowlands G, Protheroe J, Price H, Gann B, Rafi I. Health Literacy: Report from an RCGP-led health literacy workshop. Royal College of General Practitioners. 2014. Retrieved from http://www.rcgp.org.uk/news/2014/june/~/media/Files/Policy/RCGP-Health-Literacy-2014.ashx. Accessed 8 July 2016.

[12] Rootman I, Gordon-El-Bihbety D (2008). A vision for a health literate Canada.

[13] Geboers B, Brainard JS, Loke YK, Jansen CJ, Salter C, Reijneveld SA, de Winter AF (2015). The association of health literacy with adherence in older adults, and its role in interventions: a systematic meta-review. BMC Public Health.

[14] Loke YK, Hinz I, Wang X, Salter C (2012). Systematic review of consistency between adherence to cardiovascular or diabetes medication and health literacy in older adults. Ann Pharmacother.

[15] Manafo E, Wong S. Health literacy programs for older adults: A systematic literature review. Health Educ Res. 2012;27(6):947–960. 10.1093/her/cys067 22752153

[16] IROHLA Consortium. Policy Brief for Health Organisations. Health Literacy Centre Europe. November 2015. Retrieved from http://healthliteracycentre.eu/wp-content/uploads/2015/11/Brochure_Organisations.pdf. Accessed 8 July 2016.

[17] Higgins JP, Altman DG, Gøtzsche PC, Jüni P, Moher D, Oxman AD, Savović J, Schulz KF, Weeks L, Sterne JA (2011). The Cochrane Collaboration’s tool for assessing risk of bias in randomised trials. BMJ.

[18] Dattalo M, Giovannetti ER, Scharfstein D, Boult C, Wegener S, Wolff JL, Leff B, Frick KD, Reider L, Frey K (2012). Who Participates in Chronic Disease Self-Management (CDSM) programs? Differences between Participants and non-participants in a population of multi-morbid older adults. Med Care.

[19] Hussain‐Gambles M, Atkin K, Leese B (2004). Why ethnic minority groups are under‐represented in clinical trials: a review of the literature. Health Soc Care Community.

[20] McKibbon KA, Wilczynski NL, Haynes RB (2009). Retrieving randomized controlled trials from medline: a comparison of 38 published search filters. Health Inf Libr J.

[21] Dwan K, Altman DG, Cresswell L, Blundell M, Gamble CL, Williamson PR. Comparison of protocols and registry entries to published reports for randomised controlled trials. The Cochrane Library; 2011. 10.1002/14651858.MR000031.pub2 PMC7390503 21249714

[22] DeWalt DA, Hink A (2009). Health literacy and child health outcomes: a systematic review of the literature. Pediatrics.

[23] World Health Organization (2003). Adherence to long-term therapies - evidence for action.

[24] Jibaja-Weiss ML, Volk RJ, Granchi TS, Neff NE, Robinson EK, Spann SJ, Aoki N, Friedman LC, Beck JR (2011). Entertainment education for breast cancer surgery decisions: a randomized trial among patients with low health literacy. Patient Educ Couns.

[25] Smith MY, Wallace LS (2013). Reducing drug self-injection errors: a randomized trial comparing a "standard" versus "plain language" version of patient instructions for use. Res Social Adm Pharm.

[26] Australian Bureau of Statistics. A Picture of the Nation. 2006.

[27] Paasche‐Orlow MK, Parker RM, Gazmararian JA, Nielsen‐Bohlman LT, Rudd RR (2005). The prevalence of limited health literacy. J Gen Intern Med.

[28] Eckman MH, Wise R, Leonard AC, Dixon E, Burrows C, Khan F, Warm E (2012). Impact of health literacy on outcomes and effectiveness of an educational intervention in patients with chronic diseases. Patient Educ Couns.

[29] Otilingam PG, Gatz M, Tello E, Escobar AJ, Goldstein A, Torres M, Varma R (2015). Buenos Habitos Alimenticios para Una Buena Salud: Evaluation of a Nutrition Education Program to Improve Heart Health and Brain Health in Latinas. J Aging Health.

[30] Zite NB, Wallace LS (2011). Use of a low-literacy informed consent form to improve women's understanding of tubal sterilization: a randomized controlled trial. Obstet Gynecol.

[31] Kalichman SC, Cherry C, Kalichman MO, Amaral C, White D, Grebler T (2013). Randomized clinical trial of HIV treatment adherence counseling interventions for people living with HIV and limited health literacy. J Acquir Immune Defic Syndr.

[32] Reavley NJ, McCann TV, Cvetkovski S, Jorm AF (2014). A multifaceted intervention to improve mental health literacy in students of a multicampus university: a cluster randomised trial. Soc Psychiatry Psychiatr Epidemiol.

[33] Unger JB, Cabassa LJ, Molina GB, Contreras S, Baron M (2013). Evaluation of a fotonovela to increase depression knowledge and reduce stigma among Hispanic adults. J Immigr Minor Health.

[34] Giuse NB, Koonce TY, Storrow AB, Kusnoor SV, Ye F (2012). Using health literacy and learning style preferences to optimize the delivery of health information. J Health Commun.

[35] McCarthy DM, Davis TC, King JP, Mullen RJ, Bailey SC, Serper M, Jacobson KL, Parker RM, Wolf MS (2013). Take-Wait-Stop: a patient-centered strategy for writing PRN medication instructions. J Health Commun.

[36] Crowley MJ, Powers BJ, Olsen MK, Grubber JM, Koropchak C, Rose CM, Gentry P, Bowlby L, Trujillo G, Maciejewski ML (2013). The Cholesterol, Hypertension, And Glucose Education (CHANGE) study: results from a randomized controlled trial in African Americans with diabetes. Am Heart J.

[37] Duncan M, Vandelanotte C, Kolt GS, Rosenkranz RR, Caperchione CM, George ES, Ding H, Hooker C, Karunanithi M, Maeder AJ (2014). Effectiveness of a web- and mobile phone-based intervention to promote physical activity and healthy eating in middle-aged males: randomized controlled trial of the ManUp study. J Med Internet Res.

[38] Freed E, Long D, Rodriguez T, Franks P, Kravitz RL, Jerant A (2013). The effects of two health information texts on patient recognition memory: a randomized controlled trial. Patient Educ Couns.

[39] Gulliver A, Griffiths KM, Christensen H, Mackinnon A, Calear AL, Parsons A, Bennett K, Batterham PJ, Stanimirovic R (2012). Internet-based interventions to promote mental health help-seeking in elite athletes: an exploratory randomized controlled trial. J Med Internet Res.

[40] Kiropoulos LA, Griffiths KM, Blashki G (2011). Effects of a multilingual information website intervention on the levels of depression literacy and depression-related stigma in Greek-born and Italian-born immigrants living in Australia: a randomized controlled trial. J Med Internet Res.

[41] Landrey AR, Matlock DD, Andrews L, Bronsert M, Denberg T (2013). Shared decision making in prostate-specific antigen testing: the effect of a mailed patient flyer prior to an annual exam. J Prim Care Community Health.

[42] Bailey SC, Sarkar U, Chen AH, Schillinger D, Wolf MS (2012). Evaluation of language concordant, patient-centered drug label instructions. J Gen Intern Med.

[43] Calderon JL, Shaheen M, Hays RD, Fleming ES, Norris KC, Baker RS (2014). Improving diabetes health literacy by animation. Diabetes Educ.

[44] Taylor-Rodgers E, Batterham PJ (2014). Evaluation of an online psychoeducation intervention to promote mental health help seeking attitudes and intentions among young adults: Randomised controlled trial. J Affect Disord.

[45] Rosal MC, Ockene IS, Restrepo A, White MJ, Borg A, Olendzki B, Scavron J, Candib L, Welch G, Reed G (2011). Randomized trial of a literacy-sensitive, culturally tailored diabetes self-management intervention for low-income latinos: latinos en control. Diabetes Care.

[46] Horvath KJ, Trudeau SA, Rudolph JL, Trudeau PA, Duffy ME, Berlowitz D (2013). Clinical trial of a home safety toolkit for Alzheimer's disease. Int J Alzheimers Dis.

[47] Bickmore TW, Silliman RA, Nelson K, Cheng DM, Winter M, Henault L, Paasche-Orlow MK (2013). A randomized controlled trial of an automated exercise coach for older adults. J Am Geriatr Soc.

[48] Walker JG, Mackinnon AJ, Batterham P, Jorm AF, Hickie I, McCarthy A, Fenech M, Christensen H (2010). Mental health literacy, folic acid and vitamin B12, and physical activity for the prevention of depression in older adults: randomised controlled trial. Br J Psychiatry.

[49] Caldwell PH, Hamilton S, Tan A, Craig JC (2010). Strategies for increasing recruitment to randomised controlled trials: systematic review. PLoS Med.

[50] Ioannidis J (2014). Clinical trials: what a waste. BMJ.

[51] Epstein RM, Street RL (2011). The values and value of patient-centered care. Ann Fam Med.

[52] Salter C, Brainard J, McDaid L, Loke Y (2014). Challenges and opportunities: what can we learn from patients living with chronic musculoskeletal conditions, health professionals and carers about the concept of health literacy using qualitative methods of inquiry?. PLoS One.

[53] Murdoch J, Varley A, Lattimer V, Campbell J, Salter C (2014). Designing process evaluations for cluster randomised controlled trials: definitions of context and implications for data collection.

[54] Murdoch J, Salter C, Cross J, Smith J, Poland F (2013). Resisting medications: moral discourses and performances in illness narratives. Sociol Health Illn.

[55] Greenhalgh T, Snow R, Ryan S, Rees S, Salisbury H (2015). Six ‘biases’ against patients and carers in evidence-based medicine. BMC Med.

[56] Song F, Hooper L, Loke YK (2013). Publication bias: what is it? How do we measure it? How do we avoid it?. Open Access J Clin Trials.

[57] Koletsi D, Spineli LM, Lempesi E, Pandis N (2015). Risk of bias and magnitude of effect in orthodontic randomized controlled trials: a meta-epidemiological review. Eur J Orthod.

[58] Gargon E, Williamson PR, Altman DG, Blazeby JM, Clarke M (2014). The COMET Initiative database: progress and activities from 2011 to 2013. Trials.

[59] Hahn DL (2009). Importance of evidence grading for guideline implementation: the example of asthma. Ann Fam Med.

[60] Wolf MS, Bailey SC, Serper M, Smith M, Davis TC, Russell AL, Manzoor BS, Belter L, Parker RM, Lambert B (2014). Comparative effectiveness of patient-centered strategies to improve FDA medication guides. Med Care.

[61] Cordasco KM, Asch SM, Bell DS, Guterman JJ, Gross-Schulman S, Ramer L, Elkayam U, Franco I, Leatherwood CL, Mangione CM (2009). A low-literacy medication education tool for safety-net hospital patients. Am J Prev Med.

[62] Negarandeh R, Mahmoodi H, Noktehdan H, Heshmat R, Shakibazadeh E (2013). Teach back and pictorial image educational strategies on knowledge about diabetes and medication/dietary adherence among low health literate patients with type 2 diabetes. Prim Care Diabetes.

[63] Rudd RE, Blanch DC, Gall V, Chibnik LB, Wright EA, Reichmann W, Liang MH, Katz JN (2009). A randomized controlled trial of an intervention to reduce low literacy barriers in inflammatory arthritis management. Patient Educ Couns.

[64] Unk JA, Brasington R (2014). Efficacy study of multimedia rheumatoid arthritis patient education program. J Am Assoc Nurse Pract.

[65] Galliher JM, Post DM, Weiss BD, Dickinson LM, Manning BK, Staton EW, Brown JB, Hickner JM, Bonham AJ, Ryan BL (2010). Patients' question-asking behavior during primary care visits: a report from the AAFP National Research Network. Ann Fam Med.

[66] Muir KW, Ventura A, Stinnett SS, Enfiedjian A, Allingham RR, Lee PP (2012). The influence of health literacy level on an educational intervention to improve glaucoma medication adherence. Patient Educ Couns.

[67] Kripalani S, Roumie CL, Dalal AK, Cawthon C, Businger A, Eden SK, Shintani A, Sponsler KC, Harris LJ, Theobald C (2012). Effect of a pharmacist intervention on clinically important medication errors after hospital discharge: a randomized trial. Ann Intern Med.

[68] Kavin M, Anel-Tiangco RM, Mauger DT, Gabbay RA (2010). Development and pilot of a low-literacy diabetes education book using social marketing techniques.

[69] Price-Haywood EG, Harden-Barrios J, Cooper LA (2014). Comparative effectiveness of audit-feedback versus additional physician communication training to improve cancer screening for patients with limited health literacy. J Gen Intern Med.

[70] Zoellner J, Cook E, Chen Y, You W, Davy B, Estabrooks P (2013). Mixed methods evaluation of a randomized control pilot trial targeting sugar-sweetened beverage behaviors. Open J Prev Med.

[71] Brega AG, Pratte KA, Jiang L, Mitchell CM, Stotz SA, Loudhawk-Hedgepeth C, Morse BD, Noe T, Moore KR, Beals J (2013). Impact of targeted health promotion on cardiovascular knowledge among American Indians and Alaska Natives. Health Educ Res.

[72] Kiser K, Jonas D, Warner Z, Scanlon K, Shilliday BB, DeWalt DA (2012). A randomized controlled trial of a literacy-sensitive self-management intervention for chronic obstructive pulmonary disease patients. J Gen Intern Med.

[73] Goeman D, Jenkins C, Crane M, Paul E, Douglass J (2013). Educational intervention for older people with asthma: a randomised controlled trial. Patient Educ Couns.

[74] Hernandez MY, Organista KC (2013). Entertainment-education? A fotonovela? A new strategy to improve depression literacy and help-seeking behaviors in at-risk immigrant Latinas. Am J Community Psychol.

[75] Miller DP, Spangler JG, Case LD, Goff DC, Singh S, Pignone MP (2011). Effectiveness of a web-based colorectal cancer screening patient decision aid: a randomized controlled trial in a mixed-literacy population. Am J Prev Med.

[76] Jay M, Adams J, Herring SJ, Gillespie C, Ark T, Feldman H, Jones V, Zabar S, Stevens D, Kalet A (2009). A randomized trial of a brief multimedia intervention to improve comprehension of food labels. Prev Med.

[77] Zoellner J, Chen Y, Davy B, You W, Hedrick V, Corsi T, Estabrooks P (2014). Talking health, a pragmatic randomized-controlled health literacy trial targeting sugar-sweetened beverage consumption among adults: rationale, design & methods. Contemp Clin Trials.

