# Supplementary material for: Methodological review: quality of randomized controlled trials in health literacy
Source: BMC Health Serv Res. 2016 Jul 11;16:246. doi: 10.1186/s12913-016-1479-2 (PMC4940982; doi:10.1186/s12913-016-1479-2)
Supplement: Additional file 1: Table S1. — Search phrases and results. Item S2: Data extraction form. Table S3: Study characteristics. (DOCX 82 kb) [file 12913_2016_1479_MOESM1_ESM.docx]

**Table S1 Search phrases and results**

| **Type of publication** | **Pubmed** | **OVID: Embase** | **Cochrane Central** | **Cinahl (EBSCO)** | **Psychinfo EBSCO)** | **RTIPS** |
| --- | --- | --- | --- | --- | --- | --- |
|  | (randomized controlled trial[Publication Type] OR randomized[Title/Abstract]) AND  ("health-literacy"[All Fields] OR "health-literacy"[MeSH terms]) | (randomised-controlled-trial or rct).pt. or (randomised or rct).tw. or (randomised or rct).kw.  AND  (health-literacy or realm or tofhla).tw. or (realm or tofhla or health-literacy).kw. | Search terms same as pubmed | pt (randomised-controlled-trial or rct) or ti (randomized or rct) or ab (randomized or rct) or kw (randomised-controlled-trial or rct)  AND  ti (health-literacy or realm or tofhla) or ab (realm or tofhla or health-literacy) or kw (health-literacy or tofhla or realm) | Same terms as for CINAHL | “Health literacy”, searched site using Google engine |
| Total  (Unique results) | 225 | 44 not also in pubmed | 50 not already found | 3 not already found | 2 not already found | 4 not already found |
| Conf Procs or similar presentations | 2 | 18 | 6 | 0 | 0 | 0 |
| Protocols | 8 | 3 | 1 | 0 | 0 | 0 |
| Commentary or editorial | 0 | 0 | 0 | 1 | 0 | 0 |
| Remaining for title + abstract screening | 215 | 23 | 43 | 2 | 2 | 4 |

**Item S2. Data extraction form**

| **Author(s)** | **Title** | **Yr pub** | **journal** |
| --- | --- | --- | --- |
|  |  |  |  |

*Cochrane ROB, high/low/unclear = possible answers*

|  | Random sequence generation (selection bias) | Allocation concealment (selection bias) | Blinding of participants and personnel bias (performance bias) | Blinding of outcome assessment (detection bias) | Incomplete outcome data addressed (attrition bias), attrition < 20% | Risk of other bias (specify) |
| --- | --- | --- | --- | --- | --- | --- |
| Decision |  |  |  |  |  |  |
| Reasons |  |  |  |  |  |  |

*Effects of intervention (reporting bias expanded)*

| **Sample sizes in the trial arms I:C**  **(full results for)** | **Did they check that the study was adequately powered, was there a power calculation?** |
| --- | --- |
|  |  |

| **Outcomes of Interest** | **Results for intended outcomes?** | **Statis. Significant (p<0.05) benefit from the intervention for knowledge (K) or behaviour (B) or actual health outcomes (H)?** |
| --- | --- | --- |
| **Primary Outcome(s) Specified in Methods** | Specified as primary outcome or the outcome that power calculation is for. |  |
|  |  |  |
|  |  |  |
| **Other Outcomes specified in Methods** |  |  |
|  |  |  |
| **What, if Any, significant results for other outcomes? (unintended or not prespecified (with any p-values, first 5)** | | |
|  | | |

*Reported Statistics about pts (controls & intvn, patient characteristics, put NA if not reported), and who funded study:*

| **Country located** | **Study Funder** | **% pts who are not the dominant ethnic group within that country** | **% low income (<= US $20k/ yr)** | **%low + inadeq.**  **HL** | **% with < 12 yrs of eductn or GED** | **Mean age (yrs)** | **Gender % F** | **Other vulnerability features of targets (if any)** |
| --- | --- | --- | --- | --- | --- | --- | --- | --- |
|  |  |  |  |  |  |  |  |  |

*Other Study Design Features*

| **Description of Intervention and Control** | **What health condition targeted (e.g prevention or treatment)** | **Duration of obs (weeks)** | **HL instrument used (if any)** | **Comments on instrument suitability** |
| --- | --- | --- | --- | --- |
|  |  |  |  |  |

*Other comments:*

**Table S3. Study characteristics**

| **Authors, Publication year** | **Description of Intervention and Control** | **What health condition targeted (e.g. prevention or treatment)** | **Maximum duration of obs (weeks)** | **HL instrument used (if any)** |
| --- | --- | --- | --- | --- |
| Bailey et al. 2012 [1] | Intvn: Language Concordant, simplified drug regimen instructions  Control: standard drug instructions | Any (medication) | 0 (same day test) | Literacy by Karliner et al. 2008 [2] |
| Bickmore et al. 2013 [3] | Intvn: Embodied conversational agent (ECA) on a tablet computer for health promotion with supplied pedometer  Control: supplied pedometer only | Physical inactivity | 52 | Test of functional health literacy in adults (TOFHLA) [4] |
| Brega et al. 2013 [5] | Intvn: Heart health education classes over 5 evenings  Control: Not stated what education given | Cardiovascular disease and stroke: knowledge of risk factors and recognition of warning signs | 13 | Single question from Chew et al. 2004 [6]: “How confident are you filling out medical forms by yourself ?’ |
| Calderon et al. 2014 [7] | Intvn: 13 minute video, animated presentation of information  Control group: 5 pages of easy-to read diabetes information | Type 2 diabetes | 0 (same day test) | Spanish language version of TOFHLA [4] and an instrument developed by Calderon et al. = Diabetes Health Literacy Survey |
| Cordasco et al. 2009 [8] | Intvn: Low-literacy medication instruction guide (visual and written instructions)  Control: Standard written medication instructions | Any (medication) | 4 | English and Spanish versions of TOFHLA [4] |
| Crowley et al. 2013 [9] | Intvn: A nurse-administered telephone intervention incorporating CVD risk factor awareness, self-management and education and medication management facilitation  Control: Usual care comprised written educational material and standard monitoring | Cardiovascular disease among African American patients already diagnosed with T2 diabetes | 52 | Rapid estimate of adult literacy in medicine (REALM) [10] |
| Duncan et al. 2014 [11] | Intvn: Mobile phone App to monitor & remind people to eat better & exercise more, plus provided social online support.  Control: Printed information about healthy lifestyle choices | Lifestyle choices | 39 | Nutritional Literacy Survey [12] & Active Australia Survey [13] |
| Eckman et al. 2012 [14] | Intvn: Information on VHS or DVD plus printed information in booklet  Control: Booklet alone | Coronary artery disease | 26 | Rapid estimate of adult literacy in medicine (REALM) [10] |
| Freed et al. 2013 [15] | Intvn: Low reading level information leaflet  Control: Standard reading level information leaflet | Colorectal screening decisions | 0 (same day test) | Single question: ‘‘How often do you need to have someone help you when you read instructions, pamphlets, or the written material from your doctor or pharmacy?’’ |
| Galliher et al. 2010 [16] | Intvn: Ask me 3 (AM3) health communication programme introduced via brochures, posters and staff reminders  Control: Not told about AM3, asked not to change their communication behaviour with physicians for study duration | Patient question-asking behaviour with health care professional(s) | 3 | 3 questions from Chew et al. 2004 [6] |
| Giuse et al. 2012 [17] | Intvn: Routine discharge instructions in addition to instructions tailored to individual health literacy level as well as learning style (visual, aural, kinetic)  Control: Routine discharge instructions | Hypertension among patients attending emergency department | 2 | 3 questions from Chew et al. 2004 [6] and Spanish language TOFHLA [4] |
| Goeman et al. 2013 [18] | Intvn: Pamphlet and single session face-to-face in-home 1-to-1 personal education in response to patient replies on a patient-concerns-questionnaire that also instructed about inhaler technique  Control: Pamphlet only | Asthma management | 56 | None |
| Gulliver et al. 2012 [19] | Intvn: Internet delivered information about the conditions and how to get help, with multiple email reminders to review the information, with regular measurement surveys  Control: Email reminders to take measurement surveys only | Depression and/or Anxiety | 13 | Developed own instrument to assess how well patients were informed about depression and anxiety |
| Hernandez et al. 2013 [20] | Intvn: exposed to 20-30 min. fotonovela, Secret Feelings (Cabassa et al. 2010), which is written at a US 4th grade reading level and presents the story of a depressed middle age Latina mother named Sophia.  Control: Exposed to a discussion of family communication and intergenerational relationships lasting 45 min to an hour developed by the study site’s clinicians. The discussion identifies maladaptive communication patterns between immigrant parents and their US born children and offers techniques for its improvement.  Both groups then followed by 30-40 minute post-testing. | Depression | 0 (same day test) | Spanish language TOFHLA [4] |
| Horvath et al. 2013 [21] | Intvn: In addition to 15-20 minutes home education, low literacy booklet and home-safety kit (a canvas bag with low-cost sample safety items).  Control: In-home education.  Both: For 3 months, the caregivers in both arms were called biweekly by researchers to collect information on the Risky Behaviour Questionnaire (RBQ). | Prevention of accidents among dementia patients | 12 | None |
| Jay et al. 2009 [22] | Intvn: Received a Nutrition Facts Label pocket card and viewed a video explaining card use.  Control: Received standard written materials instead. | Nutrition | 0 (same day test) | Short version of TOFHLA [23] |
| Jibaja-Weiss et al. 2011 [24] | Intvn: A multi-media interactive full-narrated simple navigation low-literacy novella with relevant information.  Control: Standard printed information. | Decisions about breast cancer treatment | 56 | Developed own instrument, Incompletely described |
| Kalichman et al. 2013 [25] | Intvn-1: Flipchart standard adherence advice, pillbox practice, problem solving, additional support.  Intvn-2: Pictograph-guided adherence counselling, motivational enhancement, direct feedback, training in self-monitoring skills, medication instruction at low reading level, memory cues.  Control: General health improvement counselling, nutrition-healthy lifestyle & goals. | HIV management | 52 | TOFHLA [4] and single question from Chew et al. 2004 [6] |
| Kavin et al. 2010 [26] | Intvn-1: Low literacy Diabetes book.  Intvn-2: Low literacy Diabetes book with brief nurse tutorial.  Control: Usual care. | Type 2 diabetes management | 26 | None |
| Kiropoulos et al. 2011 [27] | Intvn: Patient + bilingual interviewer sat together in front of computer displaying educational website. For 10 minutes interviewers instructed how to use website, and patients were then given an hour to read through the online material by themselves.  Control: Interview with a bilingual interviewer who asked open-ended questions relating to the participant’s beliefs about depression including the causes, symptoms, course and development, treatments, and outcomes of depression. | Depression | 1 | D-Lit (Depression literacy) scale [28] |
| Kiser et al. 2012 [29] | Intvn: 15-30 minute standard education session and usual clinical care, in addition to one-on-one education session which utilized a literacy-sensitive handout titled Living With COPD training in inhaler technique, encouraged smoking cessation and use of a COPD action plan.  Control: 15-30 minute standard education session with usual clinical care. | Management of Chronic Obstructive Pulmonary Disease | 8 | Short version of TOFHLA [23] |
| Kripalani et al. 2012 [30] | Intvn: Counselling & medication reconciliation with telephone follow-ups from pharmacist, customised pill box, low literacy illustrated medication schedule.  Control: Treating physicians and nurses performed medication reconciliation and provided discharge counselling. | Acute coronary syndromes or decompensated heart failure | 12 | Short version of TOFHLA [23] |
| Landrey et al. 2013 [31] | Intvn: Flyer posted to patients due screening.  Control: No flyer posted. | Decisions about prostate screening | 3 | None |
| McCarthy et al. 2013 [32] | Intvn: Simple visual cue labels about when to take medication.  Control: Standard medication labelling. | Any (medication) | 0 (same day test) | REALM [10] |
| Miller et al. 2011 [33] | Intvn: Web-based CRC screening decision aid (low literacy).  Control: Touch screen, computer-based information about prescription drug refılls and safety. | Colorectal screening decisions | 24 | REALM [10] |
| Muir et al. 2012 [34] | Intvn: One-on-one session with the study coordinator, lasting approximately 20 min. which included an individualised literacy-level adapted video, and an individualised literacy-adapted brochure. Subjects received a phone call once a month as well.  Control: Standard care which involved usual management by the treating ophthalmologist, including any glaucoma education which he or she might provide. | Glaucoma management | 26 | TOFHLA [4] |
| Negarandeh et al. 2013 [35] | Intvn-1, *Pictorial image method group*. The members of this group received the education via illustrated contents within three weekly sessions, each lasting 20 min. (custom designed images multi-materials, individual sessions)  Intvn-2, *Teach back method group*. The members of this group received the education based on teach back strategy within three individual weekly sessions, each lasting 20 min.  *Usual care group*. In the diabetes clinic, an endocrinologist visited patients and prescribed medications. The routine patient education on diabetes, which the control group received, was to present an educational brochure containing information regarding diabetes control and to answer pt questions by the same community health nurse in a similar time to the intervention groups. | Adherence to diabetes medication | 6 | Persian language version of TOFHLA [4] |
| Otlingam et al. 2014 [36] | Intvn-1: 2 x 2 hour workshops on fats, taking small steps and practical information on buying healthy foods, with 30 minutes on brain health + heart health.  Intvn-2: As Intvn-1 but additional 30 minutes on heart health only.  Control – Placed on waiting list for nutritional workshops. | Nutrition, especially linked to dementia or heart disease risks | 4 | Newest Vital Signs, English or Spanish language versions [37] |
| Price-Haywood et al. 2014 [38] | Intvn: Trained primary health care professionals, in addition to audit-feedback, to improve their communication behaviours and increase cancer screening among patients with limited HL  Control: Only provided clinical performance feedback. | Cancer-screening decisions | 104 | REALM [10] |
| Reavley et al. 2014 [39] | Intvn: Emails, posters, campus events, factsheets/booklets and mental health first aid training courses.  Control: No active programme to provide similar information. | Mental illness | 104 | None |
| Rosal et al. 2011 [40] | Intvn: Usual care in addition to 12 weekly sessions (some individual, some group, between 1 & 2.5 hrs long) and a follow-up phase of 8 monthly sessions. Plus phone calls.  Control: Monitoring and usual care. | Management of T2 Diabetes | 52 | None |
| Rudd et al. 2009 [41] | Intvn: As controls but also information notebook written in plain English, booklet on getting most from doctor apt and for most patients, 2 individual appointments with an educator (usually 20 minutes long)  Control: Usual appointments with rheumatologist & notebook containing Arthritis Foundation (AF) pamphlets about their type of arthritis and about their arthritis medicines, examples of medicine calendars, and a map of the hospital. The AF materials had a reading level >= US 11th grade. | Management of rheumatoid arthritis | 52 | Arthritis-oriented version of REALM [10] |
| Smith and Wallace 2013 [42] | Intvn: Short, plain English version of instructions  Control: Standard instructions. | Injected medications | 0 (same day test) | REALM [10] |
| Taylors-Rodgers and Batterham 2014 [43] | Intvn: Received brief (3 week long) online informational programmes. The experimental condition had slightly more content than the control condition and had optional multiple-choice questions & no links to external content. The topics covered in three weeks were depression, anxiety, and suicide. Each website followed a common format: vignette of typical young person experiencing the mental health problem, description and symptoms, challenging stigmatising views, treatment, and help options.  Control: Participants were emailed links to webpages on dental hygiene, common household medications and nutrition facts. All this web content in public domain already. | Awareness of mental illness | 4 | A-Lit and D-Lit [28], Literacy of Suicide Scale [44] |
| Unger et al. 2013 [45] | Intvn: Low literacy fotonovela style booklet.  Control: Standard text pamphlet. | Depression | 4 | None |
| Unk and Brasington 2014 [46] | Invtn: Viewed multimedia presentation of material in the clinic exam room on a designated laptop computer and were given a printed copy of the slides and a CD copy of the program to review on their home computer.  Control: Sent home with standard literature about rheumatoid arthritis. | Management of rheumatoid arthritis | 4 | None |
| Walker et al. 2010 [47] | 8 arm trial, of which intervention groups received combinations of folic acid, vitamin B12, physical activity encouragement and low literacy level education on managing depression.  Control: Nutrition and pain information provided with placebo supplements. | Depression | 104 | None |
| Wolf et al. 2014 [48] | 3 Intvn groups: Exposed to different versions of low literacy medication guidelines.  Control: Conventional medication guidelines. | Any (medication) | 0 (same day test) | REALM [10] |
| Zite et al. 2011 [49] | Intvn: Low literacy sterilisation consent form. Control: Standard consent form. | Informed consent for tubal ligation (sterilisation) | 0 (same day test) | 3 questions from Chew et al. 2004 [6] |
| Zoellner et al. 2013 [50] | Intvn: 5 week education and motivational programme to reduce intake of sugary beverages.  Control: 5 week programme to encourage more physical activity. | Sugar-sweetened soft drink intake | 6 | Newest Vital Signs [37] |

Note: Intvn = Intervention group, obs = observations.

**References for Table S3.**

1. Bailey SC, Sarkar U, Chen AH, Schillinger D, Wolf MS: **Evaluation of language concordant, patient-centered drug label instructions**. *Journal of general internal medicine* 2012, **27**(12):1707-1713.

2. Karliner LS, Napoles-Springer AM, Schillinger D, Bibbins-Domingo K, Pérez-Stable EJ: **Identification of limited English proficient patients in clinical care**. *Journal of general internal medicine* 2008, **23**(10):1555-1560.

3. Bickmore TW, Silliman RA, Nelson K, Cheng DM, Winter M, Henault L, Paasche-Orlow MK: **A randomized controlled trial of an automated exercise coach for older adults**. *Journal of the American Geriatrics Society* 2013, **61**(10):1676-1683.

4. Parker RM, Baker DW, Williams MV, Nurss JR: **The test of functional health literacy in adults**. *Journal of general internal medicine* 1995, **10**(10):537-541.

5. Brega AG, Pratte KA, Jiang L, Mitchell CM, Stotz SA, Loudhawk-Hedgepeth C, Morse BD, Noe T, Moore KR, Beals J: **Impact of targeted health promotion on cardiovascular knowledge among American Indians and Alaska Natives**. *Health education research* 2013, **28**(3):437-449.

6. Chew LD, Bradley KA, Boyko EJ: **Brief questions to identify patients with inadequate health literacy**. *Health* 2004, **11**:12.

7. Calderon JL, Shaheen M, Hays RD, Fleming ES, Norris KC, Baker RS: **Improving Diabetes Health Literacy by Animation**. *The Diabetes educator* 2014, **40**(3):361-372.

8. Cordasco KM, Asch SM, Bell DS, Guterman JJ, Gross-Schulman S, Ramer L, Elkayam U, Franco I, Leatherwood CL, Mangione CM: **A low-literacy medication education tool for safety-net hospital patients**. *American journal of preventive medicine* 2009, **37**(6 Suppl 1):S209-216.

9. Crowley MJ, Powers BJ, Olsen MK, Grubber JM, Koropchak C, Rose CM, Gentry P, Bowlby L, Trujillo G, Maciejewski ML *et al*: **The Cholesterol, Hypertension, And Glucose Education (CHANGE) study: results from a randomized controlled trial in African Americans with diabetes**. *American heart journal* 2013, **166**(1):179-186.

10. Davis TC, Long SW, Jackson RH, Mayeaux E, George RB, Murphy PW, Crouch MA: **Rapid estimate of adult literacy in medicine: a shortened screening instrument**. *Family medicine* 1993, **25**(6):391-395.

11. Duncan M, Vandelanotte C, Kolt GS, Rosenkranz RR, Caperchione CM, George ES, Ding H, Hooker C, Karunanithi M, Maeder AJ *et al*: **Effectiveness of a web- and mobile phone-based intervention to promote physical activity and healthy eating in middle-aged males: randomized controlled trial of the ManUp study**. *Journal of medical Internet research* 2014, **16**(6):e136.

12. Diamond JJ: **Development of a reliable and construct valid measure of nutritional literacy in adults**. *Nutrition Journal* 2007, **6**(1):1.

13. Health AIo, Welfare: **The Active Australia Survey: A guide and manual for implementation, analysis and reporting**: Australian Institute of Health and Welfare; 2003.

14. Eckman MH, Wise R, Leonard AC, Dixon E, Burrows C, Khan F, Warm E: **Impact of health literacy on outcomes and effectiveness of an educational intervention in patients with chronic diseases**. *Patient education and counseling* 2012, **87**(2):143-151.

15. Freed E, Long D, Rodriguez T, Franks P, Kravitz RL, Jerant A: **The effects of two health information texts on patient recognition memory: a randomized controlled trial**. *Patient education and counseling* 2013, **92**(2):260-265.

16. Galliher JM, Post DM, Weiss BD, Dickinson LM, Manning BK, Staton EW, Brown JB, Hickner JM, Bonham AJ, Ryan BL *et al*: **Patients' question-asking behavior during primary care visits: a report from the AAFP National Research Network**. *Annals of family medicine* 2010, **8**(2):151-159.

17. Giuse NB, Koonce TY, Storrow AB, Kusnoor SV, Ye F: **Using health literacy and learning style preferences to optimize the delivery of health information**. *Journal of health communication* 2012, **17 Suppl 3**:122-140.

18. Goeman D, Jenkins C, Crane M, Paul E, Douglass J: **Educational intervention for older people with asthma: A randomised controlled trial**. *Patient education and counseling* 2013, **93**(3):586-595.

19. Gulliver A, Griffiths KM, Christensen H, Mackinnon A, Calear AL, Parsons A, Bennett K, Batterham PJ, Stanimirovic R: **Internet-based interventions to promote mental health help-seeking in elite athletes: an exploratory randomized controlled trial**. *Journal of medical Internet research* 2012, **14**(3):e69.

20. Hernandez MY, Organista KC: **Entertainment-education? A fotonovela? A new strategy to improve depression literacy and help-seeking behaviors in at-risk immigrant Latinas**. *American journal of community psychology* 2013, **52**(3-4):224-235.

21. Horvath KJ, Trudeau SA, Rudolph JL, Trudeau PA, Duffy ME, Berlowitz D: **Clinical trial of a home safety toolkit for Alzheimer's disease**. *International journal of Alzheimer's disease* 2013, **2013**:913606.

22. Jay M, Adams J, Herring SJ, Gillespie C, Ark T, Feldman H, Jones V, Zabar S, Stevens D, Kalet A: **A randomized trial of a brief multimedia intervention to improve comprehension of food labels**. *Preventive medicine* 2009, **48**(1):25-31.

23. Baker DW, Williams MV, Parker RM, Gazmararian JA, Nurss J: **Development of a brief test to measure functional health literacy**. *Patient education and counseling* 1999, **38**(1):33-42.

24. Jibaja-Weiss ML, Volk RJ, Granchi TS, Neff NE, Robinson EK, Spann SJ, Aoki N, Friedman LC, Beck JR: **Entertainment education for breast cancer surgery decisions: a randomized trial among patients with low health literacy**. *Patient education and counseling* 2011, **84**(1):41-48.

25. Kalichman SC, Cherry C, Kalichman MO, Amaral C, White D, Grebler T: **Randomized clinical trial of HIV treatment adherence counseling interventions for people living with HIV and limited health literacy**. *Journal of acquired immune deficiency syndromes (1999)* 2013, **63**(1):42.

26. Kavin M, Anel-Tiangco RM, Mauger DT, Gabbay RA: **Development and pilot of a low-literacy diabetes education book using social marketing techniques**. In: *Diabetes Therapy.* vol. 1; 2010: 93-102.

27. Kiropoulos LA, Griffiths KM, Blashki G: **Effects of a multilingual information website intervention on the levels of depression literacy and depression-related stigma in Greek-born and Italian-born immigrants living in Australia: a randomized controlled trial**. *Journal of medical Internet research* 2011, **13**(2):e34.

28. Griffiths KM, Christensen H, Jorm AF, Evans K, Groves C: **Effect of web-based depression literacy and cognitive–behavioural therapy interventions on stigmatising attitudes to depression**. *The British Journal of Psychiatry* 2004, **185**(4):342-349.

29. Kiser K, Jonas D, Warner Z, Scanlon K, Shilliday BB, DeWalt DA: **A randomized controlled trial of a literacy-sensitive self-management intervention for chronic obstructive pulmonary disease patients**. *Journal of general internal medicine* 2012, **27**(2):190-195.

30. Kripalani S, Roumie CL, Dalal AK, Cawthon C, Businger A, Eden SK, Shintani A, Sponsler KC, Harris LJ, Theobald C *et al*: **Effect of a pharmacist intervention on clinically important medication errors after hospital discharge: a randomized trial**. *Annals of internal medicine* 2012, **157**(1):1-10.

31. Landrey AR, Matlock DD, Andrews L, Bronsert M, Denberg T: **Shared decision making in prostate-specific antigen testing: the effect of a mailed patient flyer prior to an annual exam**. *Journal of primary care & community health* 2013, **4**(1):67-74.

32. McCarthy DM, Davis TC, King JP, Mullen RJ, Bailey SC, Serper M, Jacobson KL, Parker RM, Wolf MS: **Take-Wait-Stop: a patient-centered strategy for writing PRN medication instructions**. *Journal of health communication* 2013, **18 Suppl 1**:40-48.

33. Miller DP, Jr., Spangler JG, Case LD, Goff DC, Jr., Singh S, Pignone MP: **Effectiveness of a web-based colorectal cancer screening patient decision aid: a randomized controlled trial in a mixed-literacy population**. *American journal of preventive medicine* 2011, **40**(6):608-615.

34. Muir KW, Ventura A, Stinnett SS, Enfiedjian A, Allingham RR, Lee PP: **The influence of health literacy level on an educational intervention to improve glaucoma medication adherence**. *Patient education and counseling* 2012, **87**(2):160-164.

35. Negarandeh R, Mahmoodi H, Noktehdan H, Heshmat R, Shakibazadeh E: **Teach back and pictorial image educational strategies on knowledge about diabetes and medication/dietary adherence among low health literate patients with type 2 diabetes**. *Primary care diabetes* 2013, **7**(2):111-118.

36. Otilingam PG, Gatz M, Tello E, Escobar AJ, Goldstein A, Torres M, Varma R: **Buenos Habitos Alimenticios para Una Buena Salud: Evaluation of a Nutrition Education Program to Improve Heart Health and Brain Health in Latinas**. *Journal of aging and health* 2015, **27**(1):177-192.

37. Weiss BD, Mays MZ, Martz W, Castro KM, DeWalt DA, Pignone MP, Mockbee J, Hale FA: **Quick assessment of literacy in primary care: the newest vital sign**. *The Annals of Family Medicine* 2005, **3**(6):514-522.

38. Price-Haywood EG, Harden-Barrios J, Cooper LA: **Comparative effectiveness of audit-feedback versus additional physician communication training to improve cancer screening for patients with limited health literacy**. *Journal of general internal medicine* 2014, **29**(8):1113-1121.

39. Reavley NJ, McCann TV, Cvetkovski S, Jorm AF: **A multifaceted intervention to improve mental health literacy in students of a multicampus university: a cluster randomised trial**. *Social psychiatry and psychiatric epidemiology* 2014, **49**(10):1655-1666.

40. Rosal MC, Ockene IS, Restrepo A, White MJ, Borg A, Olendzki B, Scavron J, Candib L, Welch G, Reed G: **Randomized trial of a literacy-sensitive, culturally tailored diabetes self-management intervention for low-income latinos: latinos en control**. *Diabetes care* 2011, **34**(4):838-844.

41. Rudd RE, Blanch DC, Gall V, Chibnik LB, Wright EA, Reichmann W, Liang MH, Katz JN: **A randomized controlled trial of an intervention to reduce low literacy barriers in inflammatory arthritis management**. *Patient education and counseling* 2009, **75**(3):334-339.

42. Smith MY, Wallace LS: **Reducing drug self-injection errors: a randomized trial comparing a "standard" versus "plain language" version of Patient Instructions for Use**. *Research in social & administrative pharmacy : RSAP* 2013, **9**(5):621-625.

43. Taylor-Rodgers E, Batterham PJ: **Evaluation of an online psychoeducation intervention to promote mental health help seeking attitudes and intentions among young adults: Randomised controlled trial**. *Journal of affective disorders* 2014, **168**:65-71.

44. Batterham PJ, Calear AL, Christensen H: **The stigma of suicide scale**. *Crisis* 2013, **34**:13-21.

45. Unger JB, Cabassa LJ, Molina GB, Contreras S, Baron M: **Evaluation of a fotonovela to increase depression knowledge and reduce stigma among Hispanic adults**. *Journal of immigrant and minority health / Center for Minority Public Health* 2013, **15**(2):398-406.

46. Unk JA, Brasington R: **Efficacy study of multimedia rheumatoid arthritis patient education program**. *Journal of the American Association of Nurse Practitioners* 2014, **26**(7):370-377.

47. Walker JG, Mackinnon AJ, Batterham P, Jorm AF, Hickie I, McCarthy A, Fenech M, Christensen H: **Mental health literacy, folic acid and vitamin B12, and physical activity for the prevention of depression in older adults: randomised controlled trial**. *The British journal of psychiatry : the journal of mental science* 2010, **197**(1):45-54.

48. Wolf MS, Bailey SC, Serper M, Smith M, Davis TC, Russell AL, Manzoor BS, Belter L, Parker RM, Lambert B: **Comparative effectiveness of patient-centered strategies to improve FDA medication guides**. *Medical care* 2014, **52**(9):781-789.

49. Zite NB, Wallace LS: **Use of a low-literacy informed consent form to improve women's understanding of tubal sterilization: a randomized controlled trial**. *Obstetrics and gynecology* 2011, **117**(5):1160-1166.

50. Zoellner J, Cook E, Chen Y, You W, Davy B, Estabrooks P: **Mixed methods evaluation of a randomized control pilot trial targeting sugar-sweetened beverage behaviors**. *Open journal of preventive medicine* 2013, **3**(1):51-57.
